# The complete mitochondrial genome of *Turdus atrogularis* (Jarocki, 1819) (Aves, Passeriformes)

**DOI:** 10.1080/23802359.2025.2504601

**Published:** 2025-05-15

**Authors:** Chuan Jiang, Wenwen Zhu, Tianyi Tai, Shujia Wu, Xilong Zhao, Xuejiao Zeng, Bo Li

**Affiliations:** a College of Wildlife and Protected Area, Northeast Forestry University, Harbin, China; b School of Life Sciences, Heilongjiang University, Harbin, China; c Shenzhen Frontier Science and Innovation Research Center, Shenzhen, China; d State Forestry and Grassland Administration Detecting Center of Wildlife, Harbin, China

**Keywords:** Black-throated thrush, mitogenome, phylogeny

## Abstract

*Turdus atrogularis* is a species with a controversial taxonomic status. In this study, the first complete mitochondrial genome of *T. atrogularis* was sequenced and characterized. The mitochondrial genome (mitogenome) is 16,757 bp in length, with a higher AT content than GC content. The mitogenome consists of 13 protein-coding genes, 22 tRNA genes, 2 rRNA genes, and 1 control region, without gene rearrangements relative to the ancestral avian gene order. Phylogenetic analysis indicates a very close genetic relationship between *T. atrogularis* and *T. ruficollis*. These findings provide valuable genetic resources for understanding the classification, phylogeny, and ecological conservation of *T. atrogularis*.

## 1. Introduction

**Figure 1.**
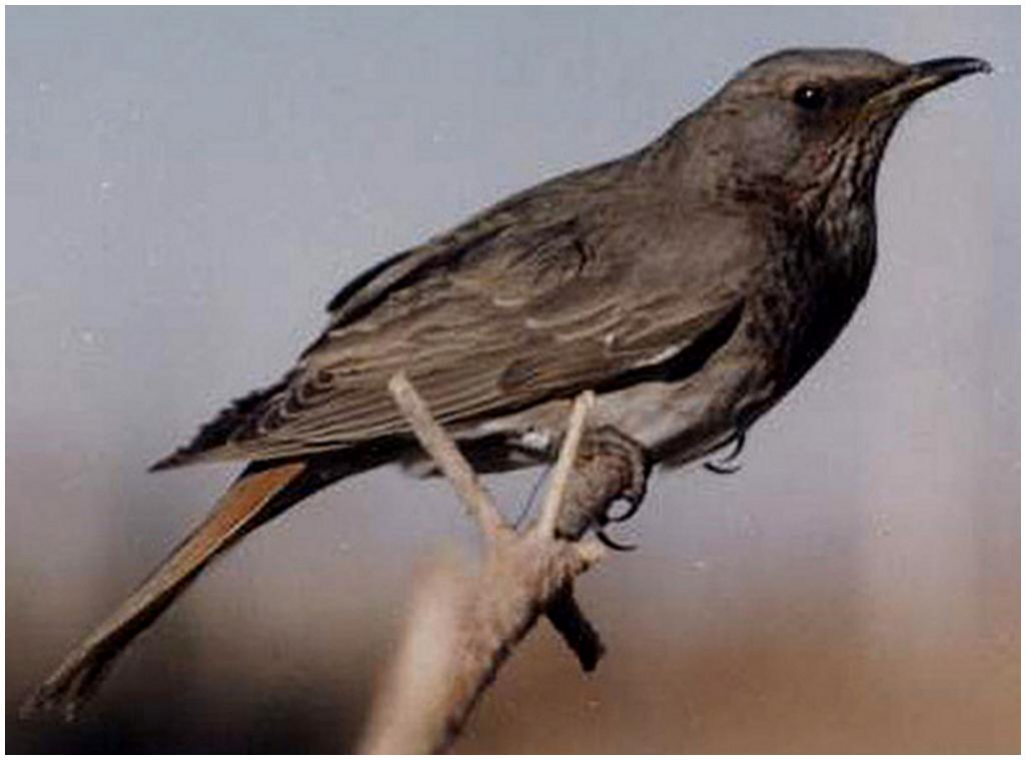
Reference photo of an adult male *turdus atrogularis*. The photo was taken in august 2024 in nenjiang, Heilongjiang, and provided by Xianda Li.

Black-throated thrush, *Turdus atrogularis* (Jarocki, 1819) (Figure 1), belonging to the order Passeriformes and the family Turdidae, is a migratory bird of the Eastern Palearctic region. Its breeding range extends from the extreme east of Europe to Western Siberia and northwest Mongolia, while its wintering range extends from the Middle East to northern Myanmar (Collar 2020). The taxonomic status of *T. atrogularis* has long been controversial, primarily in relation to red-throated thrush (*T. ruficollis*). Although the throat color of the two species is markedly different, their genetic relationship is extremely close, and they together form the red-throated and black-throated thrush complex (Knox et al. 2008; An et al. 2024). Despite previous studies using mitochondrial single-gene analysis on *T. atrogularis* and its closely related species, the limited data from a single gene makes it challenging to fully elucidate its phylogenetic relationships and genetic background (Nylander et al. 2008; An et al. 2024). The mitochondrial genome (mitogenome), as a complete carrier of linked genetic information, provides more comprehensive data support for taxonomic and phylogenetic studies (Boore 1999). Moreover, as a valuable genetic resource, it facilitates the exploration of genetic diversity within species, providing scientific basis for biodiversity conservation.

Here, we report the first complete mitogenome of *T. atrogularis*, providing new molecular evidence for its taxonomic status and phylogenetic relationships, while also offering data support for conservation biology and resource management.

## 2. Materials and methods

In March 2017, a sample of a female *T. atrogularis* from Inner Mongolia, China (42.41°N, 113.41°E), identified by densely packed dark spots near the throat, was obtained from an illegal poaching case seized by the forest police. The tissue sample is currently stored at −80 °C at the Forensic Identification Institute of Northeast Forestry University (voucher number Ta-DCW-3, contact: Bo Li, libo_770206@nefu.edu.cn). Total DNA was extracted from leg muscle tissue using a tissue extraction kit (UElandy, Suzhou, China). Subsequently, a DNA library with an insert size of approximately 350 bp was prepared according to the manufacturer’s instructions for the MGI platform, and 150 bp paired-end sequencing was performed on the DNBSEQ-T7 sequencer.

Quality filtering of raw reads was performed using fastp v0.23.4 (Chen 2023) with default parameters, resulting in clean reads. The mitogenome was assembled from the clean reads using GetOrganelle v1.7.5 (Jin et al. 2020), with the mitogenome of *Turdus ruficollis* (NC_057250) (Zhang et al. 2021) as a reference. The clean reads were mapped to the assembled mitogenome using BWA v0.7.18 (Jung and Han 2022), and site depth as well as average depth were calculated from the resulting BAM files using SAMtools v1.21 (Danecek et al. 2021). The assembled mitogenome was annotated using MITOS v2.1.9 (Zea et al. 2017) and tRNAscan-SE v1.21 (Lowe and Chan 2016), and the annotation results were manually refined using geneious v9.0.2 (Kearse et al. 2012), with reference to other published oscine mitogenomes in NCBI. The circular mitogenome map was drawn using the online tool CGView (Stothard et al. 2019).

To determine the phylogenetic position of *T. atrogularis*, we downloaded the mitogenomes of 12 *Turdus* species and two outgroup species (*Geokichla sibirica* and *Catharus fuscescens*) from NCBI. Phylogenetic analyses were conducted using maximum likelihood (ML) and Bayesian inference (BI) methods based on 13 protein-coding genes (PCGs). Sequence alignment was performed with MAFFT v7.313 (Katoh and Standley 2013), and the best substitution models were identified using PartitionFinder v2.1.1 (Lanfear et al. 2017), based on the AICc criterion (Table S1). ML analysis was conducted in IQ-TREE v1.6.8 (Nguyen et al. 2015) with 1,000 bootstrap replicates. BI analysis was carried out in MrBayes v3.2 (Ronquist et al. 2012), running for 10,000,000 generations with four chains and sampling every 1,000 generations. We discarded 25% of the initial samples as burn-in. Convergence and chain mixing were assessed in Tracer v1.6.14 (Rambaut et al. 2018), ensuring that all effective sample sizes exceeded 200. The uncorrected p-distances between *T. atrogularis* and *T. ruficollis* were calculated using MEGA11 (Tamura et al. 2021).

## 3. Result

**Figure 2.**
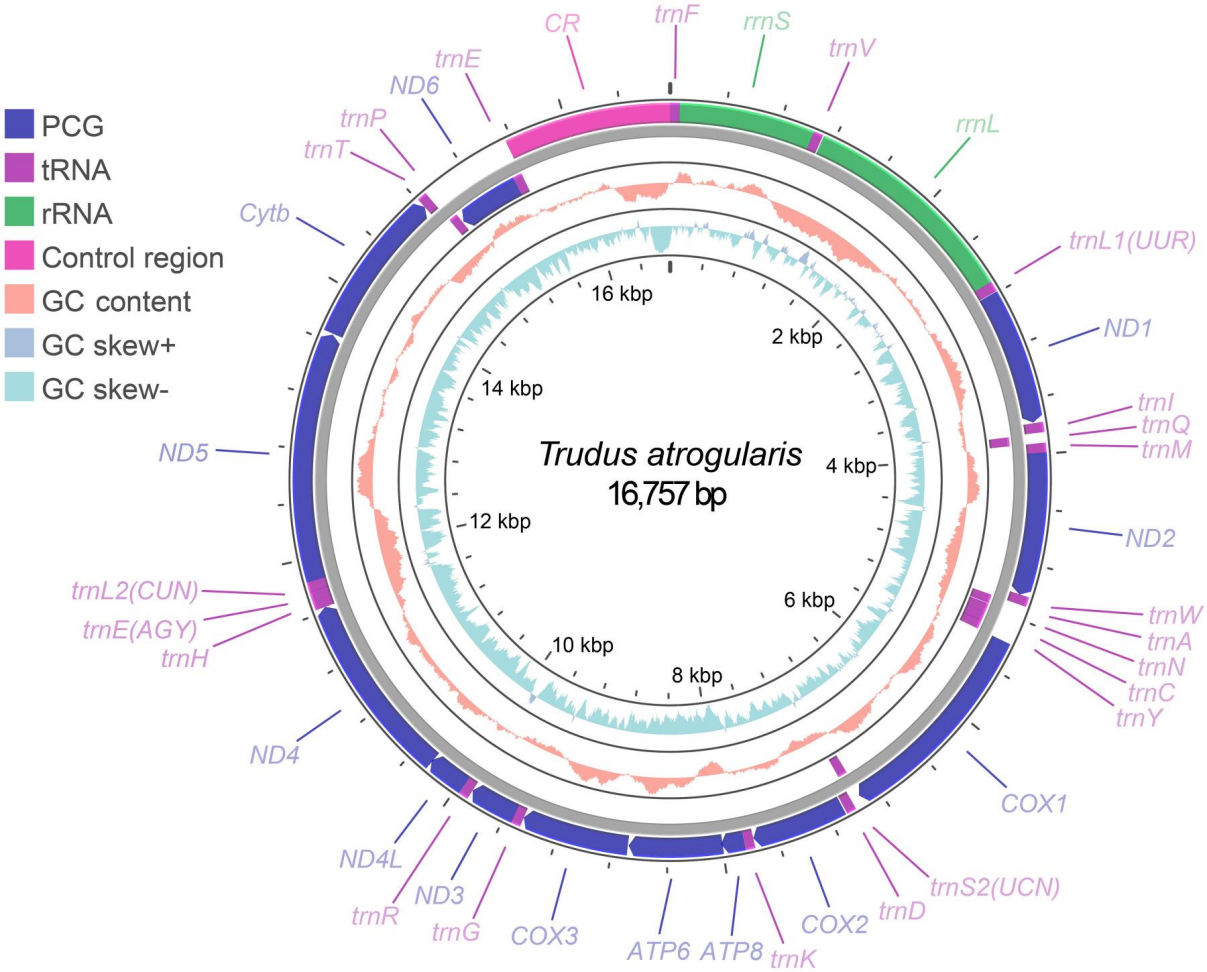
Mitogenome map of *turdus atrogularis*. GC content and GC skew were calculated using window sizes of 500 bp and 50 bp, respectively.

The assembled mitogenome of *Turdus atrogularis* was 16,757 bp in length, with an average depth of 578.934X per nucleotide (Figure S1). Annotation revealed 13 PCGs, 22 *tRNAs*, two *rRNAs*, and 1 control region, with no gene rearrangements detected, consistent with the ancestral avian gene order (Figure 2) (Gibb et al. 2007). The nucleotide composition was A: 29.31%, G:14.89%, C:32.54%, T:23.26%, with AT content (52.57%) higher than GC (47.43%). The start codons of the PCGs included ATG and GTG, while the stop codons included TAA, TAG, AGG, AGA, TA, and T. The length of *tRNAs* ranged from 66 bp to 75 bp, while the lengths of *rrnS* and *rrnL* were 977 bp and 1,590 bp, respectively.

**Figure 3.**
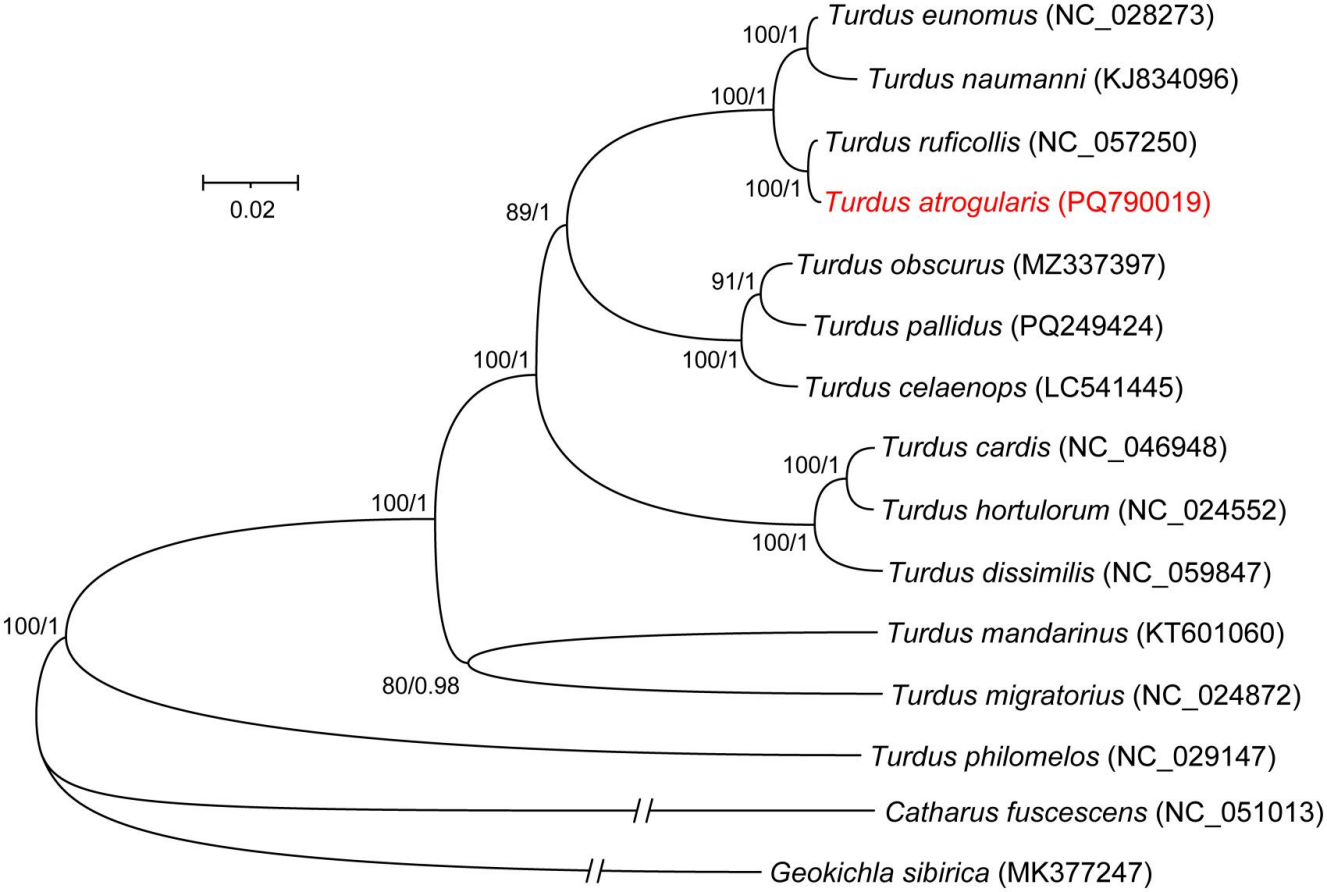
Phylogenetic tree constructed based on concatenated sequences of 13 PCGs using ML and BI methods. The tree shows branch lengths estimated using the ML method, with node support values (BP/PP) displayed next to the nodes. Outgroup branch lengths were shortened for better visualization. The species sequenced in this study was marked in red. The downloaded sequences from NCBI include *Turdus naumanni* (KJ834096) (Li et al. 2016), *Turdus celaenops* (LC541445) (unpublished), T*urdus obscurus* (MZ337397) (Zhang et al. 2021), *Turdus hortulorum* (NC_024552) (Yan et al. 2016), *Turdus migratorius* (NC_024872) (Barker 2014), *Turdus mandarinus* (KT601060) (Peng et al. 2016), *Turdus eunomus* (NC_028273) (Dong et al. 2018), *Turdus philomelos* (NC_029147) (Gibb et al. 2015), *Turdus cardis* (NC_046948) (Sun et al. 2020), *Turdus ruficollis* (NC_057250) (Zhang et al. 2021), *Turdus dissimilis* (NC_059847) (Gou et al. 2024), T*urdus pallidus* (PQ249424) (unpublished), *Catharus fuscescens* (NC_051013) (Feng et al. 2020), and *Geokichla sibirica* (MK377247) (Sun et al. 2019). The mitogenome (KT601060) is incorrectly labeled as *Gurdus merula* in NCBI and actually belongs to *Gurdus mandarinus* (see Sangster and Luksenburg 2021).

The phylogenetic tree based on the concatenated 13 PCGs constructed using ML and BI methods showed a consistent topology (Figure 3), in which *T. atrogularis* was most closely related to *T. ruficollis* (uncorrected p‐distances = 0.00378), forming a sister species relationship. These two species, in turn, were sister to *T. eunomus* and *T. naumanni*, all with the highest node support (BP = 100, PP = 1).

## 4. Discussion and conclusion

In this study, we sequenced, assembled, and annotated the mitogenome of *T. atrogularis* for the first time. The nucleotide composition and genomic structure of the mitogenome of this species exhibit characteristics similar to those of other thrushes (Sun et al. 2020; Zhang et al. 2021). Phylogenetic analysis revealed a close relationship between *T. atrogulari*s and *T. ruficollis*, with a very small genetic distance, consistent with previous findings based on a limited number of mitochondrial genes (Nylander et al. 2008; An et al. 2024). However, due to the limited information from the maternally inherited mitogenome, we still lack sufficient evidence to classify *T. atrogularis* and *T. ruficollis* as two subspecies of the same species, particularly considering the occurrence of hybridization between the two taxa, which may lead to mitogenome introgression. Therefore, further conclusions require support from population whole-genome data. Nevertheless, our study provides comprehensive genetic information from the mitogenome of *T. atrogularis*, which will serve as a valuable genetic resource for research on its speciation, phylogeny, and conservation.

## Supplementary Material

Supplemental Material

Supplemental Tables_revised.docx

## Data Availability

The mitogenome sequence can be accessed in GenBank at https://www.ncbi.nlm.nih.gov with the accession number PQ790019. The associated BioProject, BioSample, and SRA identifiers are PRJNA1200882, SAMN45916581, and SRR31789333, respectively.
