# Supplementary figures and images for: The complete mitochondrial genome of Turdus atrogularis (Jarocki, 1819) (Aves, Passeriformes)
Source: Mitochondrial DNA B Resour. 2025 May 15;10(6):485–9. doi: 10.1080/23802359.2025.2504601 (PMC12082733; doi:10.1080/23802359.2025.2504601)

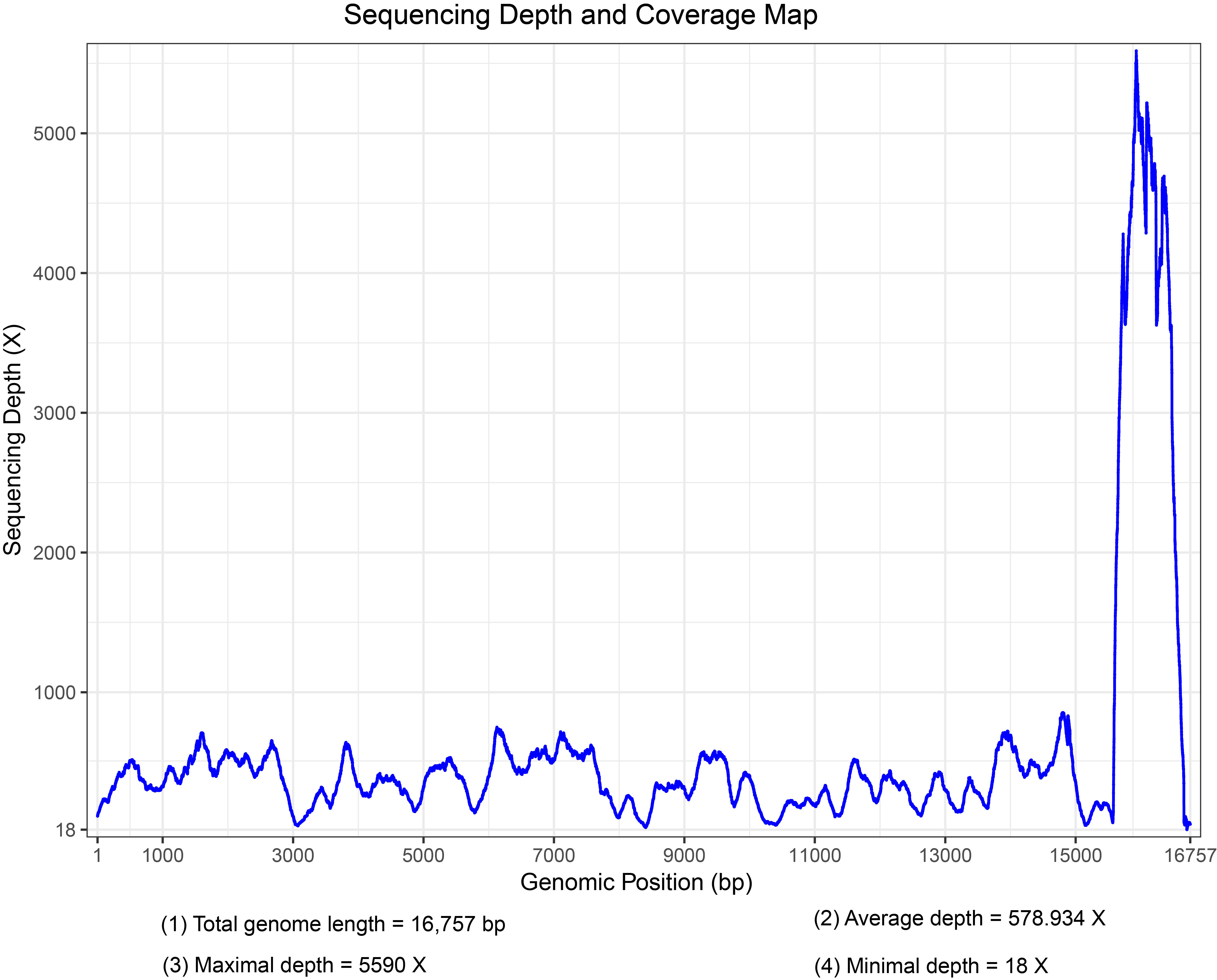

Supplement: Supplemental Material [file TMDN_A_2504601_SM5642.jpg]
