# Supplementary material for: The complete mitochondrial genome of Turdus atrogularis (Jarocki, 1819) (Aves, Passeriformes)
Source: Mitochondrial DNA B Resour. 2025 May 15;10(6):485–9. doi: 10.1080/23802359.2025.2504601 (PMC12082733; doi:10.1080/23802359.2025.2504601)
Supplement: Supplemental Tables_revised.docx [file TMDN_A_2504601_SM5730.docx]

**Table S1** The best nucleotide substitution model identified by PartitionFinder based on the AICc criterion. The values enclosed in parentheses following protein-coding genes indicate the positions of codons.

| **Software** | **Subset** | **Best Model** | **Sites** | **Subset Partitions** |
| --- | --- | --- | --- | --- |
| IQ-TREE | 1 | TVM+I+G | 903 | *ND2(1), ND4(1), ND4L(1)* |
|  | 2 | K81UF+I | 557 | *ND4(2), ND4L(2)* |
|  | 3 | K81UF+I+G | 496 | *ND3(1), Cytb(1)* |
|  | 4 | TIM+I+G | 1671 | *ATP6(1), ND4(3), ND5(3), Cytb(3)* |
|  | 5 | TRN | 55 | *ATP8(1)* |
|  | 6 | TRN+I | 1239 | *ATP6(3), ND3(3), COX1(2), Cytb(2)* |
|  | 7 | TRN+G | 344 | *ND6(1), ND6(3)* |
|  | 8 | TRN+I+G | 516 | *COX1(1)* |
|  | 9 | TIM+G | 671 | *ND1(3), ND2(3)* |
|  | 10 | HKY | 488 | *COX2(2), COX3(2)* |
|  | 11 | HKY+I | 724 | *ATP6(2), ND1(2), ND6(2)* |
|  | 12 | GTR+I | 813 | *COX3(1), ND1(1), COX2(1)* |
|  | 13 | GTR+G | 605 | *ND5(1)* |
|  | 14 | GTR+I+G | 2279 | *ATP8(2), ATP8(3), ND2(2), ND3(2), ND4L(3), ND5(2), COX1(3), COX2(3), COX3(3)* |
| MrBayes | 1 | GTR | 55 | *ATP8(1)* |
|  | 2 | GTR+I | 1347 | *ATP6(3), ND3(3), COX1(3), COX2(3), COX3(3)* |
|  | 3 | GTR+G | 1276 | *ND1(3), ND2(3), ND5(1)* |
|  | 4 | GTR+I+G | 5312 | *ATP6(1), ATP8(2), ATP8(3), ND2(1), ND2(2), ND3(2), ND4(1), ND4(2), ND4(3), ND4L(2), ND4L(3), ND5(2),ND5(3), COX1(3), COX2(3), COX3(3), Cytb(3)* |
|  | 5 | HKY | 488 | *COX2(2), COX3(2)* |
|  | 6 | HKY+I | 1620 | *ATP6(2), ND1(2), ND6(2), COX1(2), Cytb(2)* |
|  | 7 | HKY+G | 172 | *ND6(3)* |
|  | 8 | HKY+I+G | 1091 | *ND1(1), ND3(1), ND4L(1), ND6(1), Cytb(1)* |
